# A metabolic modeling approach reveals promising therapeutic targets and antiviral drugs to combat COVID-19

**DOI:** 10.1038/s41598-021-91526-3

**Published:** 2021-06-07

**Authors:** Fernando Santos-Beneit, Vytautas Raškevičius, Vytenis A. Skeberdis, Sergio Bordel

**Affiliations:** 1 Institute of Sustainable Processes, Universidad de Valladolid, Valladolid, Spain; 2 Cell Culture Laboratory, Institute of Cardiology, Lithuanian University of Health Sciences, Kaunas, Lithuania

**Keywords:** Drug discovery, Systems biology

## Abstract

In this study we have developed a method based on Flux Balance Analysis to identify human metabolic enzymes which can be targeted for therapeutic intervention against COVID-19. A literature search was carried out in order to identify suitable inhibitors of these enzymes, which were confirmed by docking calculations. In total, 10 targets and 12 bioactive molecules have been predicted. Among the most promising molecules we identified Triacsin C, which inhibits ACSL3, and which has been shown to be very effective against different viruses, including positive-sense single-stranded RNA viruses. Similarly, we also identified the drug Celgosivir, which has been successfully tested in cells infected with different types of viruses such as Dengue, Zika, Hepatitis C and Influenza. Finally, other drugs targeting enzymes of lipid metabolism, carbohydrate metabolism or protein palmitoylation (such as Propylthiouracil, 2-Bromopalmitate, Lipofermata, Tunicamycin, Benzyl Isothiocyanate, Tipifarnib and Lonafarnib) are also proposed.

## Introduction

The COVID-19 pandemic, caused by the virus SARS-CoV-2, has resulted in a substantial increase in mortality and serious economic and social disruption worldwide^1^. In this context, the rapid identification of therapeutic molecules against SARS-CoV-2 is essential. To this aim an extensive collaboration and teamwork among researchers of all academic disciplines is required^2^. Computational methods and systems biology approaches, as the one presented here, can play a significant role in this process of identification of suitable drugs.

Infection by SARS-CoV-2 has three distinct phases^3^: a first pulmonary phase in which the virus infects the host’s lung cells leading to pneumonia and acute respiratory distress syndrome, a second pro-inflammatory phase in which infected cells and lymphocytes respond by overproducing cytokines (cytokine storm), and a third prothrombic phase. In order to reduce the number of necessary hospitalizations and decrease the large pressure on healthcare systems caused by the COVID-19 pandemic, drugs searching to inhibit specifically viral proliferation during the pulmonary phase are necessary.

In order to inhibit the replication and assembly of viral particles, two main approaches can be attempted: the first one consists in targeting viral proteins involved in replication; (being the approved drug Remdesivir the best known example, which inhibits the RNA synthesis of SARS-CoV-2 by interfering with the viral RNA-dependent RNA polymerase)^4^; the second is based on the fact that many native enzymes of the host are necessary to produce the constitutive biomolecules of viral particles, which makes them potential drug targets. Some of the ways in which viruses reprogram the host metabolism and support viral replication are, among others, activation of glycolysis, fatty acid biosynthesis and elongation, protein glycosylation and inhibition of mitochondrial function^5^. It is reasonable to expect that drugs targeting key human metabolic enzymes can be used to inhibit viral replication. Indeed, nucleoside and nucleotide analogs such as Tenofovir, Sofosbuvir or Ribavirin are already being used as antiviral drugs^6–8^. Pharmacological inhibition of either acetyl-CoA carboxylase (ACC) or fatty acid synthase (FASN) has also been shown to result in a strong reduction of viral titers in vaccinia infection^9^.

In many cases, the metabolic resources of the host cell are diverted to the formation of virions via the recruitment of metabolic enzymes and their localization in “replication and transcription complexes” (RTCs), which are vesicles formed from endoplasmic reticulum (ER) membranes, in which the virus replicates. This recruitment takes place via the interaction of host enzymes with viral proteins, such as NSP3 of rotavirus, which recruits FASN, or NS5A of hepatitis C virus, which recruits phosphatidylinositol 4-kinase alpha (PI4KA)^4^. SARS-CoV-2, in the same manner as other coronaviruses, also replicates within ER derived vesicles^10^. Therefore, it is reasonable to expect that host enzymes key for viral replication are recruited to RTCs via their interaction with viral proteins. A human-SARS-CoV-2 interactome has been recently published^11^, which contains 332 high-quality human-SARS-CoV-2 protein–protein interactions, of which 90 involve metabolic enzymes. The goal of this work is to identify metabolic enzymes that are suitable drug targets against viral replication. In previous articles^12,13^ we developed the python library pyTARG, with the object of finding metabolic drug targets against cancer cell lines. This method makes use of a generic human Genome Scale Metabolic Model (GSMM), which is constrained using RNA-seq data in order to generate a context-specific model. This allows computing metabolic flux distributions and evaluating the outcomes of restricting enzymatic reaction rates. In order to use this methodology for the evaluation of the metabolic fluxes in lung cells infected by SARS-CoV-2, a literature based stoichiometric equation for the formation of virions has been added to a context-specific GSMM based on RNA-seq data of lung tissue^12,13^.

This approach provides an efficient method to identify putative drug targets against infection by SARS-CoV-2 and could be used by pharmaceutical companies in order to save considerable resources and time when developing new drugs.

## Results

### Stoichiometric equation of SARS-CoV-2 virions

The SARS-CoV-2 genome is similar to SARS-CoV, each has an Orf1ab encoding 16 predicted non-structural proteins (Nsp1-16) and each has the 4 typical coronavirus structural proteins, including: Spike (S), Envelope (E), Membrane (M) and Nucleocapsid (N). Thus, the structural information from SARS-CoV, obtained by crio-electron microscopy^14^, has been used to estimate the stoichiometric relations between the main structural proteins. Image analysis revealed a relation of 8 M protein dimers per S protein trimer in the virion surface, with an estimated number of 90 spikes per viral particle. N proteins are found both at the surface and within the virion, with M:N ratios between 1 and 3 depending on the authors^15^. The M:S ratio determined by crio-electron microscopy is equal to 5.3 and corresponds to the inverse of the ratio between the two protein lengths, which could be consistent with viral proteins being translated at the same rate per amino acid. This would lead to a M:N ratio of 1.9, which is within the previously mentioned rank and has been chosen for modeling purposes. No other proteins have been included in the structure of the virion. E protein seems to play a key role in virion assembly, but it is not present in significant quantities in viral particles. The S protein of SARS-CoV-2 has 16 N-glycosylation residues^14^ and one palmitoyl residue, while the M protein has one N-glycosylation residue and one palmitoyl residue. The lipid quantity of phospholipids per virion was estimated by considering an average virion diameter of 125 nm and assuming that the surface proteins are packed as circles touching each other forming a hexagonal pattern^15^. This geometry leaves 10% of the surface for a lipid bilayer, whose composition has been assumed to be equal to that of the human endoplasmic reticulum^16^. In a previous work^17,18^ the authors evaluated the conductance of the SARS-CoV viroporin 3a purified protein in planar lipid bilayers with a biologically relevant mix with a composition to that of intracellular organelle membranes, such as the endoplasmic reticulum (ER)-Golgi intermediate compartment (ERGIC). Thus, this assumption appears to be widely accepted.

Not only the structural proteins forming the virions are being translated, but also all the viral proteins, however only the structural proteins are being drained away from the cells when virions are secreted, the rest of the proteins are assumed to be degraded within the cell at the same rate as they are produced, and their amino acids are constantly recycled. This fact does not result in a drain of amino acids from the cells, but it has energetic costs in form of ATP and GTP. In order to have an approximate estimation of these costs, we assume that all the viral proteins are translated at a rate inversely proportional to its length. Equally, genomic RNA and sub-genomic mRNAs require methylation, which consumes SAM units. This has been modeled by assuming that the genomic and sub-genomic mRNAs are all produced in the same proportions. This choice does not have any impact on the conclusions of the article. The detailed virion composition is reported in Table 1.

**Table 1:** Composition of the viral particles used in the model of SARS-CoV-2 infected cells.

Virion components	Number of molecules per virion
Viral RNA	1
S protein	270
M protein	1440
N protein	758
Cholesterol	1600
Phosphatidylcholine	10,800
Phosphatidylethanolamine	4000
Phosphatidylinosithol	2100

### Metabolic modeling

A human Genome Scale Metabolic Model has been constrained using the *fullconstrain* function from pyTARG (https://github.com/SergioBordel/pyTARG), as described in reference^13^, in this way a context-specific model for lung cells has been obtained. The library pyTARG was previously developed to identify metabolic enzymes whose inhibition would inhibit the growth of cancer cells while having weaker effects on healthy cells. Here we follow an analogous approach in which virion production in infected cells is selectively targeted. Infection by SARS-CoV-2 has been modeled by making use of the interactions between viral proteins and human enzymes^10^, which contains 332 high-quality human-SARS-CoV-2 protein–protein interactions, of which 90 involve metabolic enzymes (see Supplementary File S1). Five of these enzymes (NDUFB9, ETFA, ATP6AP1, ATP6V1A and ATP1B1) are involved in oxidative phosphorylation. These interactions are interpreted as inhibitory or as relocating the involved proteins to RTCs instead of mitochondria, which would suppress their function. This has been modeled by constraining oxidative phosphorylation to have a zero flux in the model of infected cells. This assumption is consistent with the literature, as cells infected by viruses have shown increased lactic fermentation, in an analogous way to the Warburg effect in cancer^4^.

For the rest of metabolic enzymes expressed in the lung and interacting with viral proteins, the constraints on their rates have been removed (this is equivalent to assume that their rates could increase as a result of these enzymes being highly concentrated within RTCs). Possible changes in gene expression were modeled by identifying the transcription factors that interact with viral proteins. Only TCF12 was identified. This regulator is known to be a transcriptional repressor. Thus, the genes regulated by TCF12 are assumed to be upregulated and the constraints on their reactions were also removed in the model. The model predicts a maximal rate of virion formation of 0.0084 nmol-virions per hour and gram of dry weight of infected cells. The rate estimated by our model is consistent with data from literature^17^, which reported HIV production rates of 50,000 virions per cell and day. Assuming a cell dry weight of 0.43 ng, both results would be equivalent (i.e. this weight is of the correct size order for human cells).

The metabolic model of SARS-CoV-2 infected cells and the model of non-infected lung cells are available at https://github.com/SergioBordel/COVID-19.

Flux Balance Analysis has also been used recently to suggest putative drug targets against SARS-CoV-2^19^, leading to the identification of guanylate kinase as a potential target. The mentioned work relied on a method previously published by Aller and co-workers^20^. This approach is based on the comparison of the metabolic flux distributions under the scenarios of cell biomass and viral biomass optimization, respectively. In this approach no extra constraints are modified in the metabolic model between non-infection and infection conditions. In the approach presented here, high quality interaction data between viral and human proteins have been used to modify the constraints of the model, as it has been previously discussed. Also, our choice of putative targets has been restricted to proteins that have been confirmed to interact with viral proteins, so as to restrict the proposed drug targets to enzymes more likely involved in the infection cycle of SARS-CoV-2.

### Identification of drug targets

**Figure 1:**
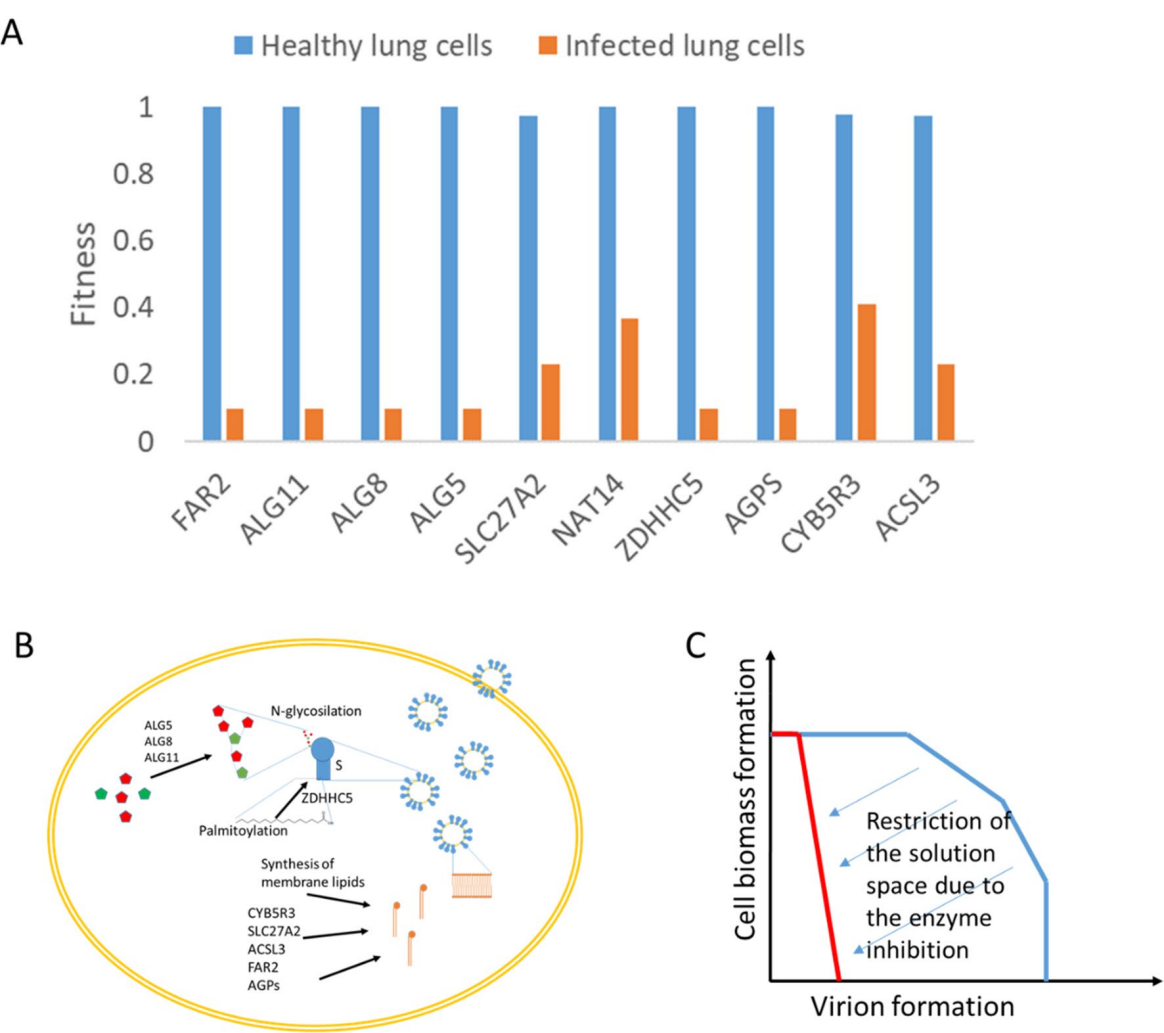
Predicted relative effects of blocking metabolic enzymes, on the healthy lung cells and the infected cells, respectively (**A**). Schema summarizing the role of the identified drug targets on the assembly of virions (**B**). Illustration of how the inhibition of an enzyme modifies the space of feasible metabolic flux distributions. The method used in this work searches to decrease the metabolic capabilities for the formation of virions, while keeping limited effects on the formation of cellular biomass (**C**).

The effects of inhibiting the function of each of the enzymes in the virus-host interactome were tested with the function *block* of pyTARG. This function restricts the rate of reactions catalyzed by the tested enzymes to 0.1 times its original value and computes the ratio by which the objective function (i.e. the rate of virion production or cellular biomass, for infected and for healthy cells, respectively) decreases. This ratio takes values from 1 (indicating no effect) and 0.1 (indicating that the objective function decreases in the same proportion as the flux in the targeted reaction). The model of non-infected lung cells was used as a way of quantifying potential secondary effects. In order to select suitable targets, a difference between both ratios (non-infected and infected) of 0.3 was used as cutoff value. The analysis was restricted to human enzymes interacting with viral proteins. Using this method, 10 human enzymes were identified (see Table 2). Predicted relative effects of targeting each enzyme in healthy and infected lung cells are shown in Fig. 1A.

**Table 2:** List of putative targets against SARS-CoV-2 replication.

Target	Viral-host interaction	Ensembl ID (human gene)	Definition	Cellular process	Known inhibitors
ALG5	Orf3a	ENSG00000120697	Dolichyl-phosphate beta-glucosyltransferase	N-Glycan biosynthesis	Celgosivir; Tunicamycin
ALG8	Orf9c	ENSG00000159063	Alpha-1,3-glucosyltransferase	N-Glycan biosynthesis	Celgosivir; Tunicamycin
ALG11	Nsp4	ENSG00000253710	Alpha-1,2-mannosyltransferase	N-Glycan biosynthesis	Celgosivir; Tunicamycin
CYB5R3	Nsp7	ENSG00000100243	NADH cytochrome-b5 reductase	Sugar metabolism	Propylthiouracil; ZINC-39395747; ZINC-05626394
ZDHHC5	Spike	ENSG00000156599	Palmitoyltransferase 5	Protein palmitoylation	2-Bromopalmitate
SLC27A2	Nsp2	ENSG00000140284	Solute carrier family 27 member 2	Lipid metabolism	Lipofermata
ACSL3	Nsp7	ENSG00000123983	Long-chain acyl-CoA synthetase	Lipid metabolism	Triacsin C
AGPS	Nsp7	ENSG00000018510	alkylglycerone phosphate synthase	Lipid metabolism	Benzyl-isothiocyanate; ZINC-69435460
FAR2	Orf9c	ENSG00000064763	alcohol-forming fatty acyl-CoA reductase	Lipid metabolism	Tipifarnib^a^; Lonafarnib^a^
NAT14	Nsp7	ENSG00000090971	putative N-acetyltransferase 14	Not determined	Not found

^a^These compounds have been identified in our docking analyses rather than in literature.

### Literature data and docking resources to identify putative inhibitors

**Figure 2:**
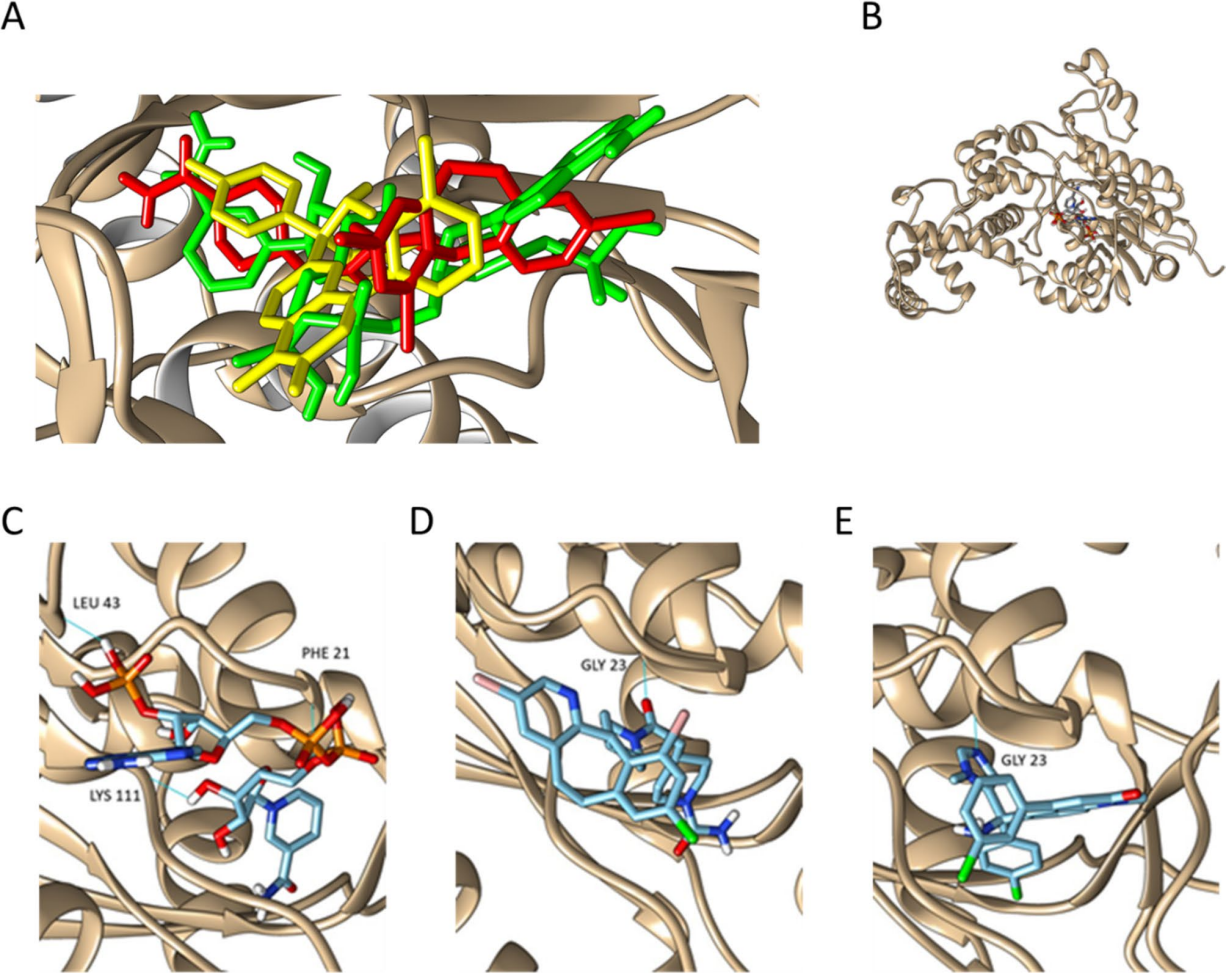
Co-factor binding site of FAR2 with the binding positions of NADPH (green) lonafarnib (red) and tipifarnib (yellow). Both drugs interact with the co-factor binding site (**A**). Fatty acyl-CoA reductase (FAR2) bound to NADPH (**B**). Docking revealed a binding affinity of − 9.5 kcal/mol for FAR2 and NADPH; one of its natural substrates (**C**), while the predicted binding affinities of Lonafarnib (**D**) and Tipifarnib (**E**) are − 10.9 kcal/mol and − 9.5 kcal/mol, respectively. This suggests that these two drugs (with a preference for Lonafarnib) could displace NADPH from its binding site and block the activity of FAR2.

A literature search using the PubMed database was carried out in order to identify inhibitors of the selected targets, and to evaluate potential secondary effects. When possible, drugs approved or under clinical trials were selected, but we also considered those in a pre-clinical status or under investigation. The literature survey yielded a total of 10 compounds (see Supplementary File S2). The affinity of these compounds for some of the target enzymes (those for which a reliable 3D structure could be obtained) was further validated using docking (see Supplementary Files S3 and S4). Docking was also used for the identification of putative inhibitors for enzymes without known inhibitors, as it is the case of Tipifarnib and Lonafarnib, predicted to bind FAR2 (Fig. 2).

Some of the 10 putative targets are known oncogenes for which drugs are already in clinical trials (*agps*, *slc27a2* and *acsl3*). The AGPS protein is inhibited, at least, by Benzyl Isothiocyanate (BITC)^21^, the anti-fungal agent Antimycin A^22^ and the investigational new drug, ZINC-69435460 [3-(2-fluorophenyl)-N-(1-(2-oxo2,3-dihydro-1H-benzo[d]imidazol-5-yl)ethyl)butanamide]^22^. Antimycin A can be discarded due to its cytotoxicity, as it is also an inhibitor of cytochrome C reductase, essential for the viability of eukaryotic cells^23^. The two other inhibitors could be promising alternatives to treat SARS-CoV-2 infections. On one hand, BITC inhibits the proliferation of human glioma U87MG and hepatic carcinoma HepG2 cells, as well as aggressive human breast tumors, by interfering with the enzymatic function of AGPS^21^. On the other hand, ZINC-69435460 makes multiple specific interactions with residues in the AGPS active site and inhibits its activity^22^. Therefore, these two compounds could be used to reduce the enzymatic function of the AGPS enzyme in the SARS-CoV-2 infected lung cells (in a similar manner as in the cancer cells), which would result in a hamper of the viral replication, while allowing the viability of the non-infected cells. The second enzyme, SLC27A2 (also known as FATP2), is a transmembrane transporter coenzyme that participates in the long-chain fatty acid beta-oxidation and peroxidase lipid metabolism^24^. In a recent publication it has been shown that the selective pharmacological inhibition of FATP2 with Lipofermata (5-bromo-5'-phenyl-spiro[3H-indole-3,2'(3'H)-[1,3,4]thiadiazol]-2(1H)-one) suppresses the activity of pathologically activated neutrophils and substantially reduces melanoma growth and invasion, while maintaining the viability of healthy cells^25^. Therefore, inhibition of this target with Lipofermata could be also a therapeutic option to treat SARS-CoV-2 infected cells. The third enzyme, ACSL3, is one of the five members of the long chain acyl-CoA synthetase (ACSL) family^26^. Specifically, ACSL3 has been identified as a host factor required for Poliovirus replication, which, as SARS-CoV-2, is a positive-sense single-stranded RNA virus that replicates its genome in association with membranes^27^. Inhibition of ACSL3 with inhibitors such as Triacsin C^28^ has been shown to reduce virion formation in human cells infected with different viruses, including Cytomegalovirus, Rotavirus and Hepatitis C^29–31^. For these reasons, and according to our metabolic modelling results, this drug constitutes a very promising therapeutic option to treat SARS-CoV-2 infection.

Other of our identified targets, ZDHHC5, is involved in the palmitoylation of proteins and has been found to interact with the Spike protein of SARS-CoV-2^11^. This post-transcriptional process plays an important role in protein–membrane interactions, protein trafficking and enzyme activity by contributing to their membrane association^32^. Palmitoylation of the SARS-CoV-2 Spike protein has been suggested to facilitate its fusion with the host cellular membrane^11^, which clearly suggests ZDHHC5 as potential target for therapeutic inhibition of SARS-CoV-2 and other coronaviruses. The literature survey has revealed some molecules that inhibit cellular processes associated with palmitoylation, with one of the best examples being 2-Bromopalmitate^33^. Other specific inhibitors of ZDHHC5 could also constitute promising drugs to combat the coronavirus infections.

**Figure 3:**
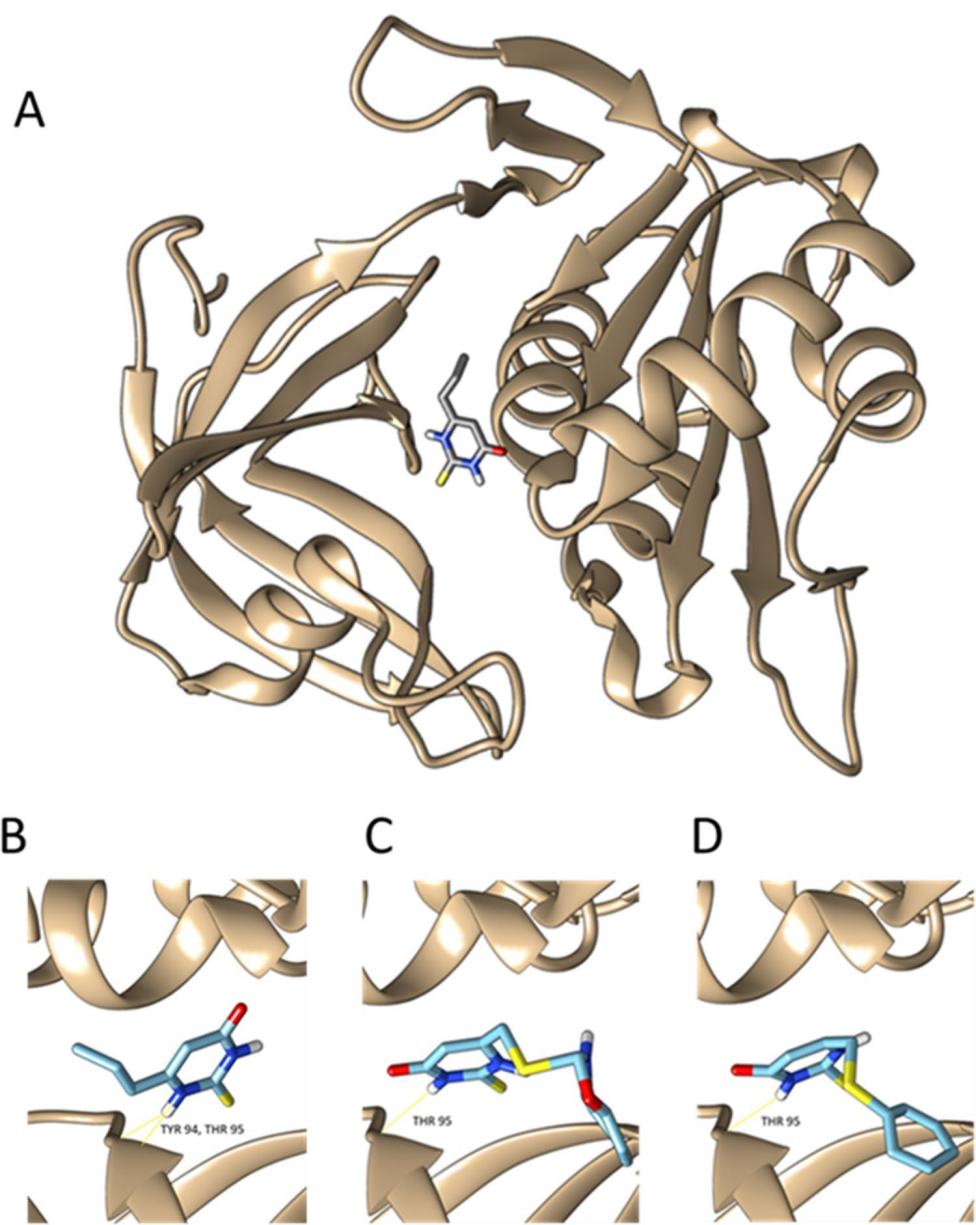
Structure of the CYB5R3 binding site of Propylthiouracil (**A**), with a binding ΔG of − 5.4 kcal/mol (**B**) and docking of ZINC39395747 (ΔG =  − 7.5 kcal/mol) (**C**) and ZINC05626394 (ΔG =  − 6.5 kcal/mol) (**D**).

Other of our identified targets, the enzyme NADH cytochrome-b5 reductase (CYB5R3), is inhibited by the approved drug, Propylthiouracil (PTU)^34^. PTU is a drug used in the therapy of hyperthyroidism and the Graves disease^35^. It is still unclear how PTU exerts its inhibitory effects on CYB5R3 at a molecular level^36^. The docking results presented in this article give an explanation of the putative inhibition mechanism, thus a repurposing strategy of PTU for COVID-19 treatment may merit investigation. Other described inhibitors of CYB5R3 are the investigational new drugs ZINC-39395747 and ZINC-05626394, which have been shown to be more potent inhibitors of CYB5R3 than PTU^37^; matching with our docking results (Fig. 3).

The rest of the identified targets are involved in N-glycan biosynthesis and glycosylation (see Table 2). Glycosylation is vital in the maturation process of viral proteins. The Spike protein of SARS-CoV-2 has 16 glycosylation residues^14^. The importance of protein glycosylation is evidenced by the use of Celgosivir (an inhibitor of N-glycan synthesis) in treating cells that harbor Dengue virus, which prevents proper protein glycosylation, leading to protein misfolding and impaired replicative efficiency^38^. Celgosivir (and also other iminosugars such as Deoxynojirimycin or Castanospermine) has been shown to be a very promising drug, not only for the treatment of Dengue virus or members of the same family (like for example ZIKV), but also for other viruses belonging to different families, such as the Hepatitis C or Influenza viruses^39–41^. As 3 of our 10 selected enzyme targets are involved in the N-Glycan biosynthesis pathway (i.e. ALG5, ALG8, ALG11), we propose treating SARS-CoV-2 with inhibitors of glycoprotein biosynthesis, such as the previous mentioned iminosugars or the nucleoside antibiotic Tunicamycin, which also inhibits N-linked protein glycosylation^42^. Interestingly, this last compound has been shown to display strong antiviral activity against certain viruses, like for example the Epstein-Barr virus^43^, and has been recently proposed as a promising therapeutic option to treat SARS-CoV-2^44^. Similarly, drugs that disrupt the integrity of the Golgi apparatus (such as Monensin, Brefeldin A, Bafilomycin and Nocodazole) also alter glycoprotein synthesis^45^ and, for this reason, could be also effective therapeutic molecules to treat COVID-19 patients. The complete list of drugs and the most relevant information regarding them, is contained in Table 3.

**Table 3:** Summary of the putative drugs against COVID-19 identified by our analysis.

Drug	Target	Company	Current use	Clinical trials (Phase)	References (PMID)	Proposed by others (PMID)
Triacsin C	Long-chain-fatty-acid-CoA ligase	–	–	–	6804425 9182714	32743584
Celgosivir	Alpha-glucosidase	Migenix	–	NCT01619969 (2) NCT00157534 (2) NCT00002150 (2)	19649930 24877997	32510142
Propylthiouracil	Thyroid peroxidase	Paladin Labs Inc	Hyperthyroidism	NCT01056419 (4)	17948378 21005687	–
2-bromopalmitate	Lipid metabolism	–	–	–	32332857 23844586	(https://doi.org/10.1101/2020.12.20.423603)
Lipofermata	Fatty acid transport proteins	–	–	–	33665270	–
Tunicamycin	UDP-HexNAc: polyprenol-P HexNAc-1-P transferases	–	–	NCT01754441 (–)	22717438	33091582
Benzyl isothiocyanate	Expression of p21/WAF1	–	–	–	17469845 16386831	–
Tipifarnib	Farnesyltransferase	Johnson and Johnson	–	NCT00093470 (3) NCT00093990 (3)	25878363 21725056	–
Lonafarnib	Farnesyltransferase	Eiger BioPharmaceuticals, Inc	HGPS	NCT00109538 (3) NCT00050336 (3) NCT03719313 (3)	17667598 23897869	–

Known targets, Literature references, clinical phase and previous suggestions by other authors as anti-COVID drugs are summarized in the table.

## Discussion

The scientific response to the COVID-19 pandemic has been unprecedented, with a huge number of studies developed in an attempt to rapidly unravel the pathogenesis of the disease and to propose potential therapeutic strategies, such as the recently approved COVID-19 vaccines or the promising treatments based on humanized monoclonal antibodies that can be used to treat the cytokine storm syndrome^46^. The severity and mortality of the disease is associated with a high level of release of cytokine in the patients, which is known as cytokine storm syndrome. IL-6 is a type of pro-inflammatory cytokine that releases in the severe COVID-19 patients. Tocilizumab, a humanized monoclonal antibody, can bind both mIL-6R (membrane bound receptor for IL-6) and sIL-6R (soluble receptor for IL-6), inhibiting its signaling pathway and stopping the cytokine storm syndrome^47^. In addition to the current developed vaccines and the humanized monoclonal antibodies, we still need more therapeutic molecules to tackle the severity and mortality of the COVID-19 disease. In this sense, the toll‐like receptor 5 (TLR‐5), which plays a vital role in various host immune defense mechanisms, can stimulate innate immune responses against common symptoms associated with COVID‐19, such as respiratory infection^48^. Another example of promising strategies to tackle the COVID‐19 outbreak is provided by an immunoinformatic analysis, which highlighted 13 Major Histocompatibility Complex‐(MHC) I and 3 MHC‐II epitopes within the spike glycoprotein of SARS‐COV‐2 that could be ideal candidates to formulate an immunogenic multi‐epitopic peptide vaccine against SARS‐COV‐2^49^.

However, if we want to decrease significantly the pressure on healthcare systems, it would be desirable to focus our efforts in finding antiviral drugs able to stop the infection by SARS-CoV-2 in its early pulmonary phase and avoid hospitalization.

The screening of other therapeutic molecules (such as antivirals) has also received substantial attention. However, finding antivirals that reduce mortality from severe respiratory viral infections has proven challenging. For example, Lopinavir-ritonavir and hydroxychloroquine are not efficient in treating COVID-19^50^. Unfortunately, one of the few antivirals that has been approved for the treatment of COVID-19, Remdesivir, has resulted not very effective for many patients^50^. These findings open a window of opportunity for antivirals that might be able to act before the fulminant inflammation caused by SARS-CoV-2.

In silico approaches, including molecular docking (one of the methods we use in this article) and molecular dynamics, have been broadly used to identify putative drugs targeting the replication cycle of SARS-CoV-2. These studies have been extensively reviewed by Sumon and co-workers^51^. The most targeted protein in these in silico approaches is the main protease (Mpro), for which 150 drugs have been suggested (50 of which approved by the FDA), followed by the spike protein (S), with 47 suggested drugs. The most suggested drug against Mpro is Lopinavir and ritonavir^52^, while gazoprevir is the most suggested drug targeting the spike protein^53^.

In this study, we have developed a metabolic modeling approach to identify human metabolic enzymes that can be targeted for therapeutic intervention against COVID-19. The model is constrained using RNA-seq data and completed with a literature based stoichiometric equation for the formation of virions. This model is used to identify metabolic reactions that, when inhibited, are expected to result in lower rates of virion production while perturbing minimally the production of cellular biomass. For example, the reactions involved in glycosylation of viral proteins, even if they carry relatively low metabolic fluxes (compared to other reactions such as those in glycolysis) are good targets because they are essential for protein activity.

Along with the predictions of metabolic enzymes of the host, that could serve as targets for therapeutic intervention against COVID-19, we have utilized docking analysis and an in depth revision of the scientific literature to propose a list of drug candidates. In total, we have proposed 12 bioactive molecules that could be promising drugs to treat COVID-19 patients. Among the proposed therapeutic molecules, those in clinical trials or approved, such as Tunicamycin and PTU, constitute the best options to investigate. We have also identified Triacsin C as very promising drug to tackle SARS-CoV-2. This compound has been shown to be very effective against different viruses, including some positive-sense single-stranded RNA viruses, such as the Picornavirus^28–31^. Similarly, we have also identified the drug Celgosivir, in clinical trials^54^, what was proven against cells infected with different types of viruses, such as Dengue, Zika, Hepatitis C and Influenza^38–41^. Other drugs targeting proteins of lipid metabolism, carbohydrate metabolism or protein palmitoylation (such as Bromopalmitate, Lipofermata, Benzyl Isothiocyanate, Tipifarnib and Lonafarnib) are also proposed in this study. The work also provides a systematic schema for target/drug screenings that allows investigators to seek them directly. The testing of some of those compounds may encourage others to do the same with other investigational molecules, expanding the options to find drugs with therapeutic value for the treatment of different types of coronaviruses infections.

In conclusion, the optimism surrounding the word wide COVID-19 vaccination program should not deflect from the search for an effective therapeutic option against the virus. Systematic validation of different approved drugs, compounds in clinical trials or in a preclinical stage, including the ones proposed in this study, will be key to find important COVID-19 treatment options in selected patient groups.

## Materials and methods

### Metabolic modeling

A human genome scale metabolic model has been constrained using the *fullconstrain* function from pyTARG (https://github.com/SergioBordel/pyTARG), creating a context-specific metabolic model for lung cells. The way in which pyTARG uses RNA-seq data to constrain GSMMs is described in reference^13^. The library pyTARG requires the installation of the library COBRApy and is compatible with the version 0.9.0 of COBRApy (solver glpk).

A maximal value is given to the rates of each of the reactions in the model, this maximal rate is set based on the expression level of the genes encoding the associated enzymes measured in Reads Per Million and Kilo-base (RPMK). Reactions not associated to any gene are left unconstrained. The proportionality constant between gene expression and maximal rate is purely phenomenological and has been chosen to reproduce the experimental growth rate of the cancer cell line A549. The chosen proportionality constant was 0.0027 mmol g-DW^−1^ h-^−1^ per unit of RPMK. The accuracy of the metabolic flux predictions obtained by pyTARG has proven to be much higher than that of previously proposed methods such as PRIME^55^. This is mostly due to the fact that pyTARG imposes constraints in almost all the metabolic reactions of the model.

The GSMM utilized is an updated version of the HMR human metabolic model^56^. This model can also be downloaded in SBML format together with the pyTARG library. The lung RNA-seq data necessary to constrain the model were obtained from the Human Protein Atlas (www.proteinatlas.org)^57^. In order to model infection by SARS-CoV-2, oxidative phosphorylation has been constrained to have a zero rate. Attenuation of respiration is known to be a general feature of viral infection^5^. In our case, this is further supported by the fact that 5 genes involved in oxidative phosphorylation (NDUFB9, ETFA, ATP6AP1, ATP6V1A and ATP1B1) are part of the human-SARS-CoV-2 interactome^11^. These interactions are interpreted as inhibitory or as relocating the involved proteins to RTCs instead of mitochondria, which would suppress their function. For the rest of metabolic enzymes expressed in lung and interacting with viral proteins, the constraints on their rates have been removed (this is equivalent to assume that their rates could increase as a result of these enzymes being highly concentrated within RTCs). The list of human transcription factors and the genes regulated by them were obtained from the TRANSFAC database^58^. The metabolic model of SARS-CoV-2 infected cells and the model of non-infected lung cells are available at https://github.com/SergioBordel/COVID-19. Both models are in SBML format and can be used to compute distributions of metabolic fluxes using the function *flux* from pyTARG.

### Identification of drug targets

The effects of blocking metabolic enzymes were modeled by selecting virion production as objective function and using the function *block* from pyTARG. This function reduces the flux in the selected metabolic reaction to 0.1 times its original value and computes the ratio by which virion production is reduced as a result of this restriction. In order to evaluate the effects on non-infected cells, a lung metabolic model was used and production of cellular biomass was chosen as an objective function.

### Calculation of protein structures

Three dimensional structures of the target proteins were generated using the SWISS-MODEL cloud based modeling tool^59^ in the cases of: O00116 (template PDB ID 4BC7), O95573 (template PDB ID 5MSS), Q2TAA5 (template PDB ID Q2TAA5), Q8WUY8 (template PDB ID 5L7J), Q9Y673 (template PDB ID 5MLZ), O14975 (template PDB ID 3A9V) and Q9C0B5 (template PDB ID 6BMS). Q9BVK2 was modelled with I-TASSER^60^. Q96K12 was modelled with Rosetta^61^. QMEAN was used to assess homology model quality^62^.

### Docking

Q8IVS2 (PDB ID 2C2N) and P00387 (PDB ID 1UMK) structures were acquired directly from RCSB PDB (https://www.rcsb.org)^63^. As other human structures were not available, homology models were used instead. Protein model structures were prepared initially with UCSF Chimera^64^ and then converted to pdbqt file format with Open Babel^65^. Cofactor biding sites on PDB structures and templates were used as potential docking locations to set up cubic docking calculations. The calculations were performed in boxes of 20 Å^3^. The conformer models of the ligands were acquired from PubChem^66^ or ZINC^67^ databases and then converted to pdbqt file format with Open Babel^65^. QuickVina-W was used to carry out docking task with all prepared structural data^68^. Docking results were visualized, analyzed and imaged with UCSF Chimera^64^.

Thermodynamic affinities for the natural substrates of the enzymes and the tested ligands can be found in supplementary file S4.

## Supplementary Information

Supplementary Information 1.

Supplementary Information 2.

Supplementary Information 3.

Supplementary Information 4.
