# Supplementary material for: A metabolic modeling approach reveals promising therapeutic targets and antiviral drugs to combat COVID-19
Source: Sci Rep. 2021 Jun 7;11:11982. doi: 10.1038/s41598-021-91526-3 (PMC8184994; doi:10.1038/s41598-021-91526-3)

Supplementary figures for:

A metabolic modeling approach reveals promising therapeutic targets and antiviral drugs to combat COVID-19

Fernando Santos-Beneit[1], Vytautas Raškevičius[2], Vytenis A. Skeberdis[2], Sergio Bordel[1,2].

1. Institute of Sustainable Processes, Universidad de Valladolid, Spain.
2. Cell Culture Laboratory, Institute of Cardiology, Lithuanian University of Health Sciences, Kaunas, Lithuania

* Sergio Bordel. **Email:** sergio.bordel@uva.es

List of proposed drugs

Celgosivir
PubChem CID: 60734
Molecular Formula: C12H21NO5

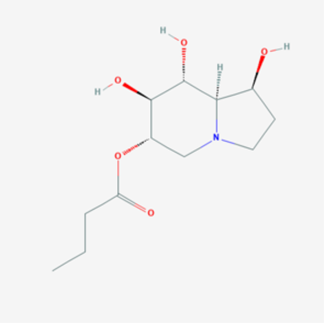

Tunicamycin
PubChem CID: 57654701
Molecular Formula: C37H60N4O16

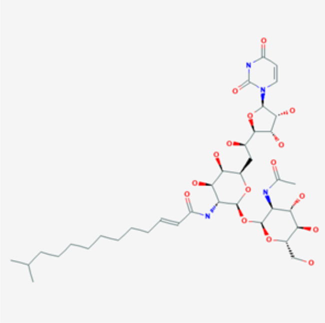

Propylthiouracil
PubChem CID: 657298
Molecular Formula: C7H10N2OS

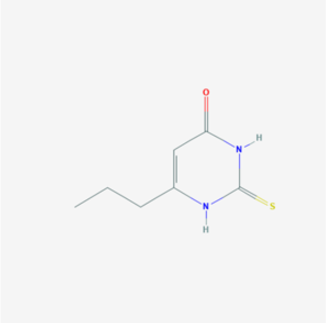

Dehydro-ZINC39395747
PubChem CID: 702583
Molecular Formula: C12H9N3O2S2

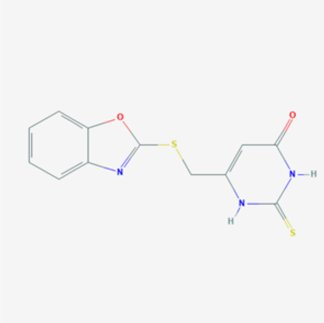

2-Bromohexadecanoic acid
PubChem CID: 82145
Molecular Formula: C16H31BrO2

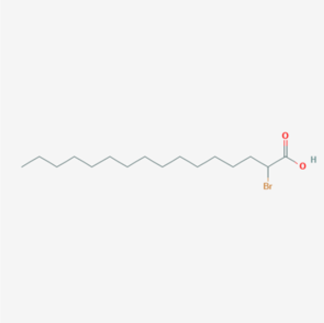

Lipofermata
PubChem CID: 3136622
Molecular Formula: C15H10BrN3OS

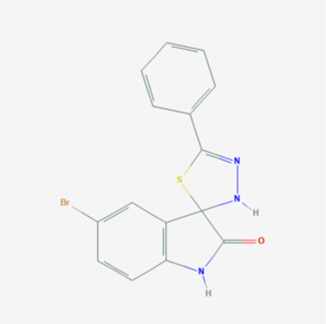

Triacsin c
PubChem CID: 9576787
Molecular Formula: C11H17N3O

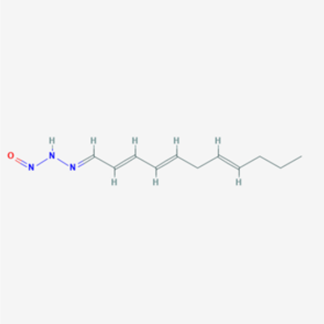

Benzyl isothiocyanate
PubChem CID: 2346
Molecular Formula: C8H7NS

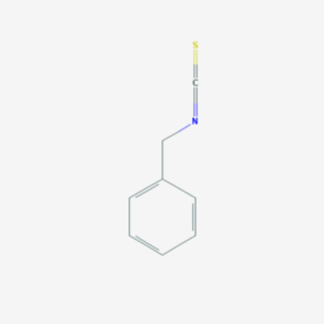

Tipifarnib
PubChem CID: 159324
Molecular Formula: C27H22Cl2N4O

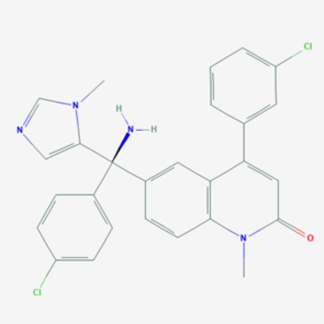

Lonafarnib
PubChem CID: 148195
Molecular Formula: C27H31Br2ClN4O2

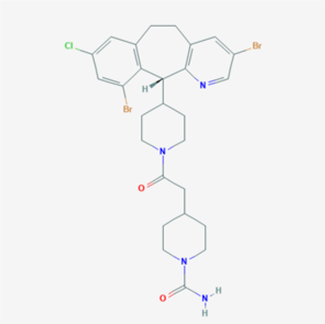

Supplement: Supplementary file 2 — Supplementary Information 2. [file 41598_2021_91526_MOESM2_ESM.pdf]
