# Supplementary material for: A metabolic modeling approach reveals promising therapeutic targets and antiviral drugs to combat COVID-19
Source: Sci Rep. 2021 Jun 7;11:11982. doi: 10.1038/s41598-021-91526-3 (PMC8184994; doi:10.1038/s41598-021-91526-3)
Supplement: Supplementary file 3 — Supplementary Information 3. [file 41598_2021_91526_MOESM3_ESM.pdf]

Supplementary material

A metabolic modeling approach reveals promising therapeutic targets and antiviral drugs to combat COVID-19

Fernando Santos-Beneit[1], Vytautas Raškevičius[2], Vytenis A. Skeberdis[2], Sergio Bordel[1,2].

1. Institute of Sustainable Processes, Universidad de Valladolid, Spain.
2. Institute of Cardiology, Lithuanian University of Health Sciences, Kaunas, Lithuania

* Sergio Bordel. **Email:** sergio.bordel@uva.es

Fernando Santos-Beneit: https://orcid.org/0000-0001-7986-5557

Sergio Bordel: https://orcid.org/0000-0001-6162-6478

Table S1. Composition of the viral particles used in the model of SARS-CoV-2 infected cells.

Virion components	Number of molecules per virion
Viral RNA	1
S protein	270
M protein	1440
N protein	758
Cholesterol	1600
Phosphatidylcholine	10800
Phosphatidylethanolamine	4000
Phosphatidylinosithol	2100

Docking results:

In order to quantify the interaction between each target enzyme and its putative inhibitors, the ΔG of binding was calculated after docking. The results are shown in the following table.

Table S2. Target-inhibitor binding affinities predicted by docking.

Gene	Uniprot ID	Ihibitor	CID	affinity (kcal/mo)
FAR2	Q96K12	Lonafarnib	148195	-10.9
FAR2	Q96K12	Tipifarnib	159324	-9.5
ALG8	Q9BVK9	Castanospermine	54445	-5.5
CYB5R3	P00387	Propylthiouracil (PTU)	657298	-5.4
CYB5R3	P00387	ZINC39395747	-	-7.5
CYB5R3	P00387	ZINC05626394	-	-6.5
ACSL3	O95573	Triacsin C	9576787	-6.1
SLC27A2	O14975	Diclofenac	3033	-7.2
ZDHHC5	Q9C0B5	2-bromopalmitate	82145	-4.6

The most negative values of ΔG correspond to the inhibitors of FAR2, Lonafarnib and Tipifarnib, which bind in the same region as NADPH with equal or higher affinity (see the main figure).

Propyltyouracil is a drug already approved against hyperthyroidism, thus it can be particularly interesting for further research. However, the experimental compounds ZINC39395747 and ZINC05626394 have a stronger binding to their substrate CYB5R3. Figure 1 shows the docking results for these three compounds.

List of proposed drugs:

Celgosivir
PubChem CID: 60734
Molecular Formula: C12H21NO5

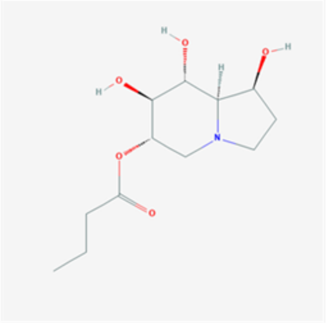

Tunicamycin
PubChem CID: 57654701
Molecular Formula: C37H60N4O16

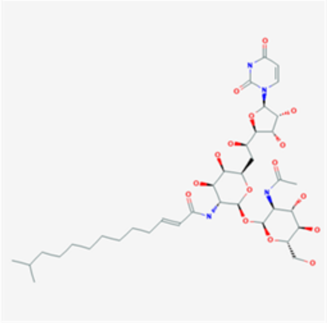

Propylthiouracil
PubChem CID: 657298
Molecular Formula: C7H10N2OS

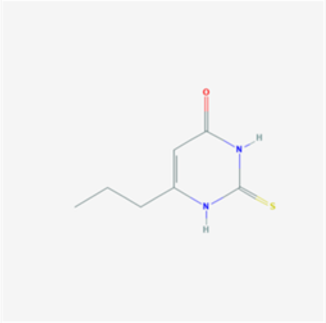

Dehydro-ZINC39395747
PubChem CID: 702583
Molecular Formula: C12H9N3O2S2

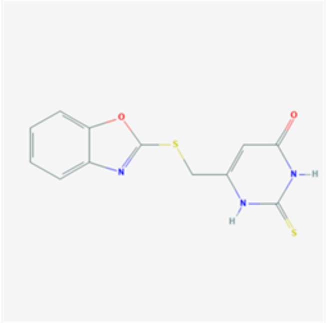

2-Bromohexadecanoic acid
PubChem CID: 82145
Molecular Formula: C16H31BrO2

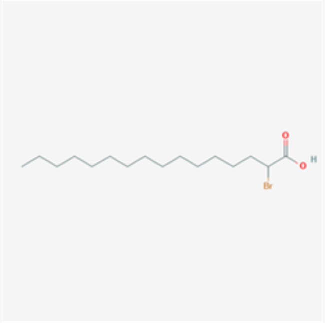

Lipofermata
PubChem CID: 3136622
Molecular Formula: C15H10BrN3OS

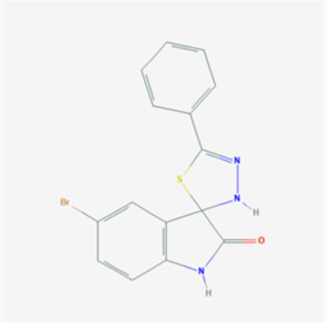

Triacsin c
PubChem CID: 9576787
Molecular Formula: C11H17N3O

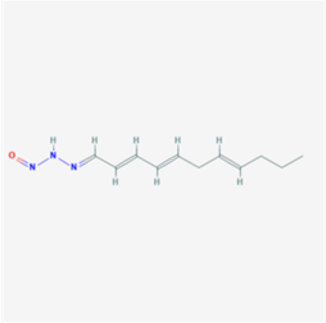

Benzyl isothiocyanate
PubChem CID: 2346
Molecular Formula: C8H7NS

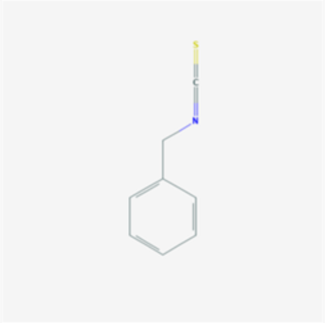

Tipifarnib
PubChem CID: 159324
Molecular Formula: C27H22Cl2N4O

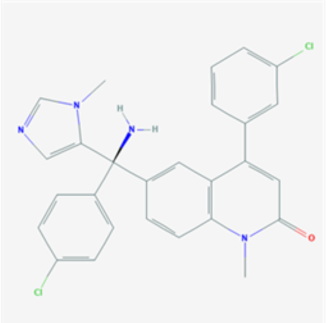

Lonafarnib
PubChem CID: 148195
Molecular Formula: C27H31Br2ClN4O2

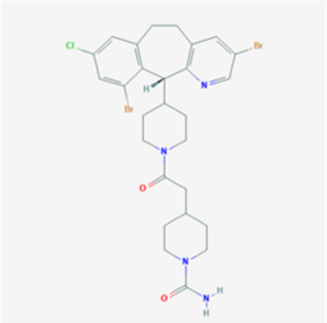

Figure S1. Structures and indentifiers of each of the putative inhibitors identified.
